# Supplementary material for: Covalent organic frameworks with high quantum efficiency in sacrificial photocatalytic hydrogen evolution
Source: Nat Commun. 2022 Apr 29;13:2357. doi: 10.1038/s41467-022-30035-x (PMC9054748; doi:10.1038/s41467-022-30035-x)
Supplement: Supplementary file 1 — Supplementary Information [file 41467_2022_30035_MOESM1_ESM.pdf]

Supplementary information

Covalent organic frameworks with high quantum efficiency in sacrificial photocatalytic hydrogen evolution

Chunzhi Li,[1,2] Jiali Liu,[1,2] He Li,[1,*] Kaifeng Wu,[3] Junhui Wang,[3,*] Qihua Yang[1,*]

[1] State Key Laboratory of Catalysis, Dalian Institute of Chemical Physics, Chinese Academy of Sciences, 457 Zhongshan Road, Dalian 116023, China.

[2] University of Chinese Academy of Sciences, Beijing 100049, China.

[3] State Key Laboratory of Molecular Reaction Dynamics, Dalian Institute of Chemical Physics, Chinese Academy of Sciences, 457 Zhongshan Road, Dalian 116023, China.

[*] Email addresses: lihe@dicp.ac.cn, wjh@dicp.ac.cn, yangqh@dicp.ac.cn.

Supplementary Methods.

Materials and instrumentation

All chemicals used in this study were of analytical grade and used as received unless otherwise specified.

Thermal gravimetric analyses (TGA) were performed on a NETZSCH STA 449F3 analyzer, with a temperature range from 30 to 100 °C and a heating rate of 10 °C min^{-1} in air atmosphere. Powder x-ray powder diffraction (PXRD) patterns were measurement on a Rigaku RINT D/Max-2500 powder diffraction system, equipped with Cu Kα radiation (λ = 1.54 Å). Transmission electron microscopy (TEM) images were collected using a HITACHI HT7700 microscope at an acceleration voltage of 100 kV. High-resolution transmission electron microscopy (HRTEM) analysis was performed using a JEM-2100 microscope. Scanning electron microscopy (SEM) characterizations were collected using JSM-7900F. Atomic force microscopy (AFM) analysis was performed using Nano Wizard Ultra Speed &inVia Raman. Dynamic light scattering (DLS) experiments were performed using Zetasizer Nano. Nitrogen sorption characterization was performed on an automatic volumetric adsorption analyzer (Micromeritics ASAP2020). Before analysis, all the samples were carefully degassed at 100 °C for 6 hours under the vacuum of 10 mmHg. The BET surface area was calculated from the adsorption data in a relative partial pressure (P/P_0) range of 0.04 to 0.20. The total pore volume was calculated at P/P_0 of 0.99 using a single-point adsorption value. The pore diameter was determined from the adsorption branch by using the Non-Localized Density Functional Theory (NLDFT) method. The Fourier-transform infrared spectroscopy (FT-IR) spectra were recorded from 400-4000 cm^{-1} on a Nicolet Nexus 470 IR spectrometer by using KBr pellets. All samples were dried with an infrared lamp before analysis. Solid-state ^{13}C CP-TOSS NMR spectra were performed on a Bruker 600 MHz spectrometer. ^{13}C and signals were referenced to tetramethylsilane (TMS). UV/Vis absorption spectra were recorded on SHIMADZU UV-Vis 2550 spectrophotometer.

Mott-Schotty plots and measurement was performed using a CH Instruments Model 760E electrochemical work station (CHI760E). The as-prepared materials were dispersed in a mixture of ethanol and Nafion (5 wt% in ethanol) by sonication and then casted onto the pre-treated glassy carbon electrode (GCE) surface. The Mott-Schotty plots measurements were conducted in a three-electrode single cell, with the COF/GCE

as the working electrode, a Pt plate as the counter electrode, and a saturated mercury electrode (SCE) as the reference electrode. The electrolyte was deionized water containing with 0.20 M Na_2SO_4.

Structural modeling of CYANO-COF

The process of simulating COF structure was performed by the Materials Studio software. The hexagonal lattice with P6/M symmetry group was set as the initial eclipsed COF structure. After the smallest asymmetric fragment was filled into the blank cell, the Forcite tools package was employed to optimize the cell geometry including energy minimization. The AB stacking structure was built with the similar process as described above, with the exception that a supercell with double c value was selected as the initial cell of staggered structure. The cell optimized from the Universal force fields was subsequently refined using the Pawley refinement method in Reflex tools.

Computational method of charge distribution

We have employed the VASP[1,2] to perform all the spin-polarized density functional theory (DFT) calculations within the generalized gradient approximation (GGA) using the Perdew-Burke-Ernzerhof (PBE)[3] formulation. We have chosen the projected augmented wave (PAW) potentials[4] to describe the ionic cores by using a plane wave basis set with a kinetic energy cutoff of 450 eV, and the valence electrons were taken into account. Partial occupancies of the Kohn−Sham orbitals were allowed using the Gaussian smearing method and a width of 0.05 eV. The electronic energy was considered self-consistent when the energy change was smaller than 10^{-5} eV. A geometry optimization was considered convergent when the energy change was smaller than 0.03 eV/Å. The brillouin zone is sampled with 1 × 1 × 1 Gamma mesh[5].

AQE measurement

The AQE was measured using a 300 W Xe lamp (PLS-FX300, Perfectlight) with different band-pass filters of 400, 450, 500, 550, 600 and 650 nm. The number of incident photons reaching the solution was measured using a calibrated Si photodiode (LS-100, EKO Instruments Co., LTD). The numbers of photons were counted according to a litrature method[6] using photon-to-current conversion with a Si photodiode and a multimeter in the device shown in Supplementary Figure 25. The AQE was calculated using the following equation:

$$\text{AQE} = \frac{2 \times number\ \ of\ \ evolved\ \ H_2\ \ molecules}{number\ \ of\ \ incident\ \ photons} \times 100\%$$

The numbers of photons were measured at various positions by sliding the position of a Si photodiode with an interval of 3 mm, the total incident photons can be then calculated by numerically integrating the obtained data over the entire light acceptance area (7.5 cm ×7.5 cm) of the reactor.[6] The total numbers of incident photons at the wavelength of 400, 450, 500, 550 600 and 650 nm were summarized in Supplementary Figure 25.

Photocatalytic hydrogen evolution with thin CYANO-CON film

The thin CYANO-CON film deposited on glass support was prepared as follows. 2 mg of CYANO-CON powder (deposited with 1 wt% Pt) and 200 µL Nafion (5 wt% in ethanol) were added into ethanol (1 mL), and the mixture was sonicated for 1 h. After sonication, a colloidal solution was formed. Thin CYANO-CON film was prepared by drop-casting the solution onto glass with a rough surface. The resulting film was dried at 120 ºC for 6 h. For the hydrogen evolution experiment using CYANO-CON film, the CYANO-CON coated glass was immersed in a flask with quartz filter containing 0.1 M ascorbic acid water solution (100 mL). The resulting mixture was degassed by Ar bubbling for 30 minutes. The reaction system was irradiated with a 300 W Xe lamp (PLS-FX300, Perfectlight) for the time specified using cut-on filters (λ > 420 nm) without stirring. Gas samples were taken with a gas-tight syringe (Hamilton 1700) and the HER rate was analyzed by GC.

Photocatalytic oxygen evolution

A flask with quartz filter was charged with CYANO-CON (20 mg), a certain amount of $Co(NO_3)_2$ as a co-catalyst, 100 mL water containing 0.5 mmol $AgNO_3$, and 100 mg of La_2O_3 as pH buffer agent. The mixture was sonicated for 10 min and the solution was evacuated several times to completely remove air. The reaction was then illuminated with a 300 W Xe light source for the time specified using cut-on filters (λ > 420 nm) under reduced pressure. Gas samples were run on a gas chromatograph (Agilent 8860) equipped with Molecular Sieve 5A column connected to thermal conductivity detector, referencing against standard gas with a known concentration of oxygen.

Supplementary Figures and Tables.

Supplementary Table 1. Fractional atomic coordinated for unit cell of CYANO-COF (AA stacking) calculated using the Materials Studio modeling program after performing the Pawley Refinement.

Space group	P6/M		
Calculated cell parameters	a = b = 28.781 Å, c = 3.5954 Å, α = β = 90°, γ = 120°, R_p = 3.99%, R_{wp} = 4.91%		
atoms	x	y	z
C1	0.72406	0.36341	0.5
C2	0.6946	0.39125	0.5
O3	0.71734	0.44005	0.5
C4	0.7756	0.38743	0.5
N5	0.81128	0.44314	0.5
C6	0.86599	0.46069	0.5
C7	0.88424	0.42341	0.5
C8	0.93572	0.43926	0.5
C9	0.9725	0.49294	0.5
C10	0.95542	0.53154	0.5
C11	0.90218	0.51541	0.5
C12	0.88604	0.55566	0.5
N13	0.87327	0.58791	0.5
H14	0.79033	0.36108	0.5
H15	0.80037	0.47236	0.5
H16	0.86264	0.38054	0.5
H17	0.94414	0.40698	0.5
H18	0.98086	0.57429	0.5

Supplementary Table 2. Fractional atomic coordinated for unit cell of CYANO-COF (AB stacking) calculated using the Materials Studio modeling program after performing the Pawley Refinement.

Space group		P63/M	
Calculated cell parameters		a = b = 28.781 Å, c = 7.191 Å, α = β = 90°, γ = 120°	
atoms	x	y	z
C1	1.05739	0.03008	0.25
C2	1.02794	0.05792	0.25
O3	1.05067	0.10672	0.25
C4	1.10893	0.05409	0.25
N5	1.14462	0.10981	0.25
C6	1.19932	0.12735	0.25
C7	1.21757	0.09008	0.25
C8	1.26905	0.10593	0.25
C9	1.30583	0.1596	0.25
C10	1.28876	0.19821	0.25
C11	1.23551	0.18207	0.25
C12	1.21937	0.22232	0.25
N13	1.2066	0.25458	0.25
H14	1.12367	0.02774	0.25
H15	1.1337	0.13902	0.25
H16	1.19597	0.0472	0.25
H17	1.27747	0.07365	0.25
H18	1.3142	0.24095	0.25
C19	0.60928	0.30325	0.25
C20	0.63873	0.27541	0.25
O21	0.616	0.22661	0.25
C22	0.55774	0.27924	0.25
N23	0.52205	0.22353	0.25
C24	0.46735	0.20598	0.25
C25	0.44909	0.24325	0.25
C26	0.39761	0.2274	0.25

C27	0.36083	0.17373	0.25
C28	0.37791	0.13512	0.25
C29	0.43115	0.15126	0.25
C30	0.4473	0.11101	0.25
N31	0.46007	0.07876	0.25
H32	0.543	0.30559	0.25
H33	0.53296	0.19431	0.25
H34	0.47069	0.28613	0.25
H35	0.3892	0.25968	0.25
H36	0.35247	0.09238	0.25

Supplementary Table 3. The textural parameters of COFs.

Sample	Surface area ($m^2\ g^{-1}$)	Pore volume ($cm^3\ g^{-1}$)	Pore size (nm)
BD-COF	519	0.39	1.0-2.5
BD-CON	472	0.37	0.7, 1.0-2.5
CYANO-COF	559	0.60	1.0-2.5
CYANO-CON	356	0.28	1.0-2.5

Supplementary Table 4. Hydrogen evolution performance for CYANO-COF using different scavengers.[a]

Scavenger	Ascorbic acid (0.1 M)	Sodium ascorbate (0.1 M)	Na_2SO_3 (0.1 M)	TEOA (10 vol.%)
HER (μmol h^{-1})	1217	104	8.35	17.2

[a] Reaction conditions: 20 mg of CYANO-COF was suspended in 100 mL of aqueous solution of the sacrificial donor (ascorbic acid, sodium ascorbate, Na_2SO_3 or TEOA) with corresponding concentrations, irradiated by a 300 W Xe lamp (λ > 420 nm).

Supplementary Table 5. Wavelength-dependent AQE of photocatalytic H_2 production

for CYANO-CON without co-catalyst.

λ (nm)	400	450	500	550	600	650
H_2 evolution rate (μ mol h^{-1})	8.98	17.4	21.3	20.1	5.75	0.99
Number of photons (x 10^{20} h^{-1})	4.14	7.71	9.58	13.7	13.3	11.6
AQE (%)	2.61	2.72	2.67	1.77	0.52	0.10

Supplementary Table 6. Comparison of photocatalytic hydrogen evolution performances of CYANO-CON with representative COFs and COF-based composites.

Photocatalyst	λ (nm)	Sacrificial reagent	Reaction parameters	Co-cat.	HER (μmol h^{-1})	HER (μmol g^{-1} h^{-1})	AQE %	Ref.
CYANO-CON	>420	AA	20 mg CON, 0.1 M AA (100 mL)	Pt (1 wt%)	2684	134200	82.6 (at 450 nm)	This work
CYANO-COF	>420	AA	20 mg COF, 0.1 M AA (100 mL)	Pt (1 wt%)	1217	60850	-	This work
NKCOF-108	>420	AA	10 mg COF, 0.1 M AA (100 mL)	Pt (5 wt%)	120	11600	2.96 (at 520 nm)	[7]
CTF-HUST-A1	>420	TEOA	50 mg COF, 10 mL TEOA, 90 mL H_2O	Pt (3 wt%)	460	9200	7.4 (at 420 nm)	[8]
Py-ClTP-BT-COF	>420	AA	20 mg COF, 0.1 M AA (50 mL)	Pt (5 wt%)	177.5	8875	8.45 (at 420 nm)	[9]
FS-COF+WS5F	>420	AA	5 mg COF, 0.1 M AA (25 mL)	Pt (8 wt%)	81.5	16300	~7.2 (at 420 nm)	[10]
g-$C_{18}N_3$-COF	>420	AA	50 mg COF, 1 M AA (100 mL)	Pt (3 wt%)	14.6	292	1.06 (at 420 nm)	[11]
sp^{2}c-COF$_{ERDN}$	>420	TEOA	50 mg COF, 10 mL TEOA, 90 mL H_2O	Pt (3 wt%)	106	2120	0.46 (at 420 nm)	[12]
TpPa-COF$(CH_3)_2$	>420	Sodium ascorbate (SA)	10 mg COF, 50 mL PBS buffer (pH = 7)	Pt (3 wt%)	83.3	8330	-	[13]
CTF-HUST-C1	>420	TEOA	50 mg COF, 10 mL TEOA, 90 mL H_2O	Pt (3 wt%)	255	5100	-	[14]

g-$C_{40}N_3$-COF	>420	TEOA	50 mg COF, 10 mL TEOA, 100 mL H_2O	Pt (3 wt%)	206	4120	4.84 (at 420 nm)	[15]
g-$C_{54}N_6$-COF	>420	TEOA	50 mg COF, 10 mL TEOA, 100 mL H_2O	Pt (3 wt%)	126	2519	-	[16]
ter-CTF-0.7	>420	TEOA	50 mg COF, 10 mL TEOA, 90 mL H_2O	Pt (2 wt%)	966	19300	22.8 (at 420 nm)	[17]
ZnPor-DETH-COF	>400	TEOA	2.5 mg COF, 5 mL PBS buffer (pH = 7), TEOA (50 μL)	Pt (8 wt%)	1.03	413	0.063 (at 450 nm)	[18]
Tp-2C/BPy^{2+}-COF	>420	AA	10 mg COF, 0.1 M AA (100 mL)	Pt (3 wt%)	346	34600	6.93 (at 420 nm)	[19]
TpPa-Cl_2	>420	SA	10 mg COF, 50 mL PBS buffer (pH = 7)	Pt (3 wt%)	76	7600	17.0 (at 400 nm)	[20]
TtaTfa	>420	AA	3 mg COF, 0.1 M AA (16 mL)	Pt (8 wt%)	62.1	20700	1.43 (at 450 nm)	[21]
CdS-CTF-1	>420	Lactic acid (LA)	20 mg photocatalyst, 8 mL LA, 80 mL H_2O	Pt (1 wt%)	228.6	11430	16.3 (at 420 nm)	[22]
TiO_2-TaPa-1-COF	>420	SA	10 mg photocatalyst, 50 mL PBS buffer (pH = 7)	Pt (3 wt%)	111.9	11190	7.6 (at 420 nm)	[23]
g-C_3N_4-COF	>420	TEOA	10 mg photocatalyst, 20 mL TEOA, 180 mL H_2O	Pt (2 wt%)	1005.8	10058	20.7 (at 425 nm)	[24]
MoS_2/TpPa-1-COF	>420	AA	10 mg photocatalyst, 100 mg AA, 50 mL H_2O	-	55.9	5590	0.76 (at 420 nm)	[25]
CdS-COF	>420	LA	30 mg photocatalyst, 1 mL LA, 9 mL H_2O	Pt (0.5 wt%)	110.3	3678	4.2 (at 420 nm)	[26]
Pt-PVP-TP-COF	>420	AA	10 mg photocatalyst, 950 mg AA, 100 mL H_2O	Pt (6 wt%)	84.2	8420	0.4 (at 475 nm)	[27]
CNS-COF	>420	TEOA	20 mg photocatalyst, 20 mL TEOA, 180 mL H_2O	Pt (3 wt%)	928	46400	31.8 (at 425 nm)	[28]
30%PEG@BT-COF	>420	AA	10 mg photocatalys	Pt (3.5 wt%)	111.4	11140	11.2 (at	[29]

			t, 0.1 M AA (100 mL)				420 nm)	
COF-CN	>420	SA	30 mg photocatalyst, 50 mL PBS buffer (pH = 7)	Pt (3 wt%)	384	12800	15.1 (at 500 nm)	[30]
TiO_2/TbBd	>380	TEOA	10 mg photocatalyst, 1 mL TEOA, 9 mL H_2O	Pt (3 wt%)	39.6	3962	-	[31]

Supplementary Table 7. Comparison of photocatalytic hydrogen evolution performances of CYANO-CON with representative polymer and polymer-based composites.

Photocatalyst	λ (nm)	Sacrificial reagent	Reaction parameters	Co-cat.	HER (μmol h^{-1})	HER (μmol g^{-1} h^{-1})	AQE %	Ref.
CYANO-CON	>420	AA	20 mg CON, 0.1 M AA (100 mL)	Pt (1 wt%)	2684	134200	82.6 (at 450 nm)	This work
CYANO-COF	>420	AA	20 mg COF, 0.1 M AA (100 mL)	Pt (1 wt%)	1217	60850	-	This work
PyBS-3	>420	AA	10 mg photocatalyst, 10 mL DMF, 1 M AA (90 mL)	Pt (3 wt%)	430	43000	29.3 (at 420 nm)	[32]
PyBS-3	>300	AA	10 mg photocatalyst, 10 mL DMF, 1 M AA (90 mL)	Pt (3 wt%)	1050	105000	-	[32]
S-CMP-3	>420	TEA	25 mg photocatalyst, H_2O : TEA :MeOH (1:1:1 vol, 25 mL)	Pd (0.7 wt%)	77.7	3106	13.2 (at 420 nm)	[33]
P10	>420	TEA	25 mg photocatalyst, H_2O : TEA :MeOH (1:1:1 vol, 25 mL)	Pd (0.4 wt%)	81.5	3260	11.6 (at 420 nm)	[34]
PDBTSO	>420	TEOA	10 mg photocatalyst, 20 mL TEOA, 80 mL H_2O, 2 mmol K_2HPO_4	Pt (3 wt%)	442	44200	-	[35]
TxPP1@T-10	Uv-vis	MeOH	100 mg photocatalyst, 100 mL MeOH, 900 mL H_2O	Pt (1 wt%)	2194.5	21945	-	[36]
P10-e	>420	MeOH/TEA	2.5 mg photocatalyst, H_2O :	Pd (0.4 wt%)	36.3	14520	20.4 (at 420	[37]

			TEA :MeOH (1:1:1 vol, 25 mL)				nm)	
TiO_2@BpZn-COP	>420	TEOA	20 mg photocatalyst, 15 mL TEOA, 85 mL H_2O	Pt (3 wt%)	2682.6.6	1333	2.5 (at 420 nm)	[38]
PDBTSO@TiO_2	>420	TEOA	10 mg photocatalyst, 20 mL TEOA, 80 mL H_2O	Pt (3 wt%)	515	51500	13 (at 420 nm)	[39]
P3HT-g-C_3N_4	>500	AA	10 mg photocatalyst, 1 M AA (10 mL)	Pt (1 wt%)	3045	304500	77.4 (at 420 nm)	[40]
Co-S/g-C_3N_4	AM1.5G	TEOA	5 mg photocatalyst, 6 mL TEOA, 44 mL H_2O	-	51.6	10329	-	[41]
α-Fe_2O_3/g-C_3N_4	>400	TEOA	10 mg photocatalyst, 10 mL TEOA, 90 mL H_2O	Pt (3 wt%)	314	31400	44.4 (at 420 nm)	[42]
CN-L0.10	>420	TEOA	10 mg photocatalyst, 5 mL TEOA, 45 mL H_2O	Pt (3 wt%)	22.3	2235	4.8 (at 420 nm)	[43]
g-C_3N_4 nanomesh	>420	TEOA	10 mg photocatalyst, 10 mL TEOA, 90 mL H_2O	Pt (3 wt%)	85.1	8510	5.1 (at 420 nm)	[44]
GD-C_3N_4	>420	TEOA	10 mg photocatalyst, 10 mL TEOA, 100 mL H_2O	Pt (3 wt%)	230.6	23060	31.1 (at 420 nm)	[45]

Supplementary Table 8. Oxygen evolution performance of COF by using different additives.[a]

COFs	Additives		O_2 evolution rate
	La_2O_3	$Co(NO_3)_2$	(μmol/h)
-	+	+	0
CYANO-CON	-	-	0
CYANO-CON	+	-	0
CYANO-CON	+	+	1.9
BD-CON	+	+	0.05
BD-CON	+	-	0

[a] Reaction conditions: 20 mg of COFs was suspended in 100 mL of an aqueous solution with different additive (100 mg La_2O_3, 1wt % $Co(NO_3)_2$) and 0.5 mmol $AgNO_3$ as sacrificial agent, irradiated by a 300 W Xe lamp (λ > 420 nm).

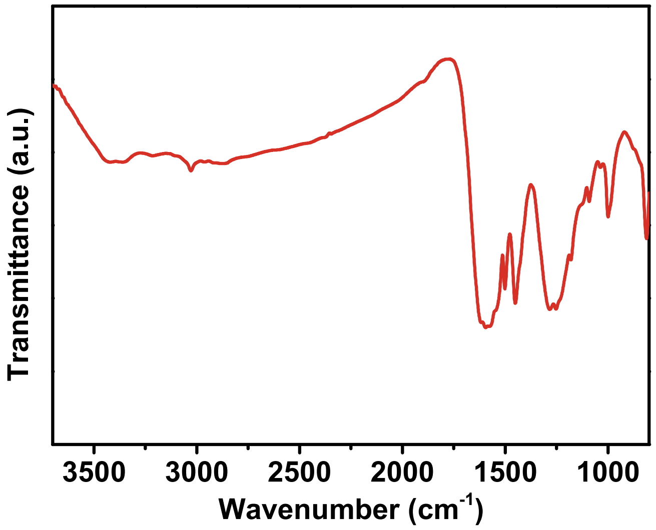

Supplementary Figure 1. FT-IR characterization. FT-IR spectrum of BD-COF.

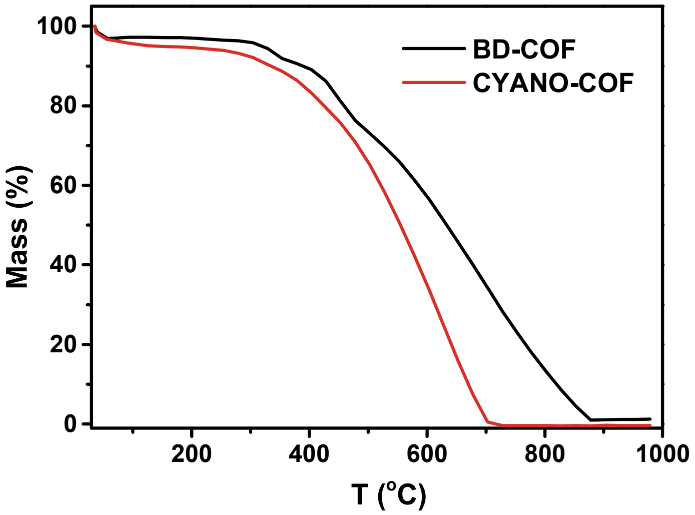

Supplementary Figure 2. TG analysis. TG curves of BD-COF and CYANO-COF under air atmosphere.

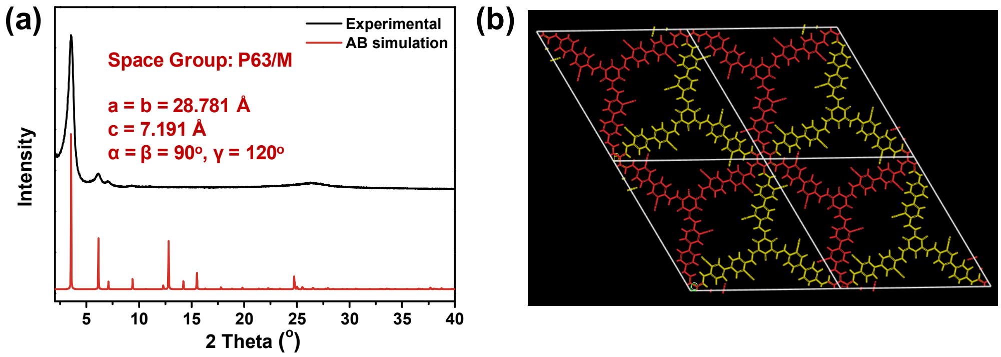

Supplementary Figure 3. Crystalline structure of CYANO-COF. (a) Simulated PXRD pattern of staggered models and (b) reproduced AB stacking crystal structure of CYANO-COF.

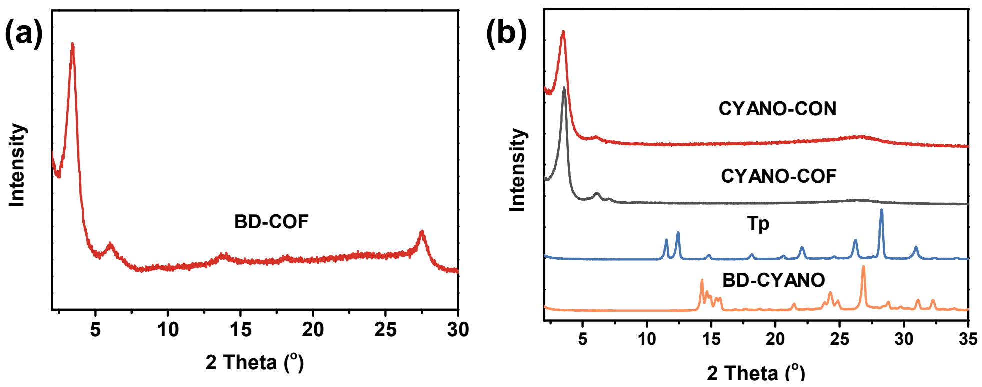

Supplementary Figure 4. PXRD analysis of COFs and monomers. PXRD patterns of (a) BD-COF, (b) CYANO-CON, CYANO-COF, Tp and BD-CYANO.

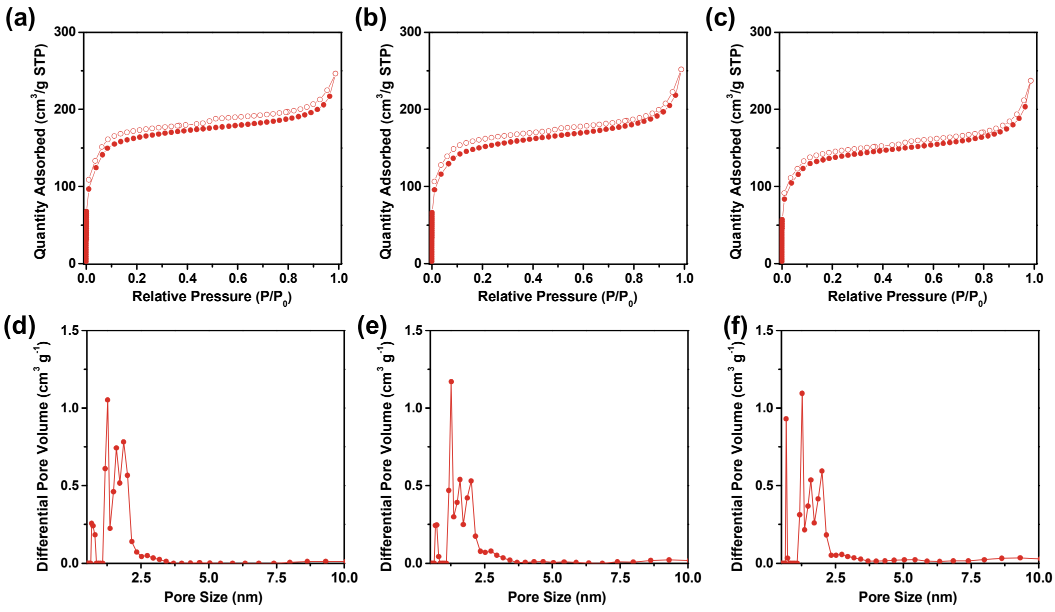

Supplementary Figure 5. N_2 adsorption isotherms and pore size distributions. (a-c) N_2 adsorption and desorption isotherms and (d-f) pore size distributions calculated by NLDFT of (a,d) CYANO-COF, (b,e) BD-COF and (c,f) BD-CON.

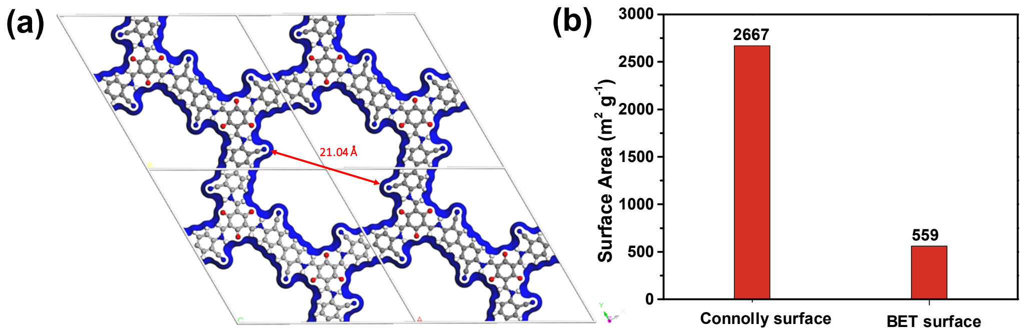

Supplementary Figure 6. Calculated Connolly surface area of CYANO-COF. (a) Top view of calculated Connolly surface and predicted pore size of CYANO-COF with AA stacking model, (b) comparison of Connolly surface area and experimental BET surface area.

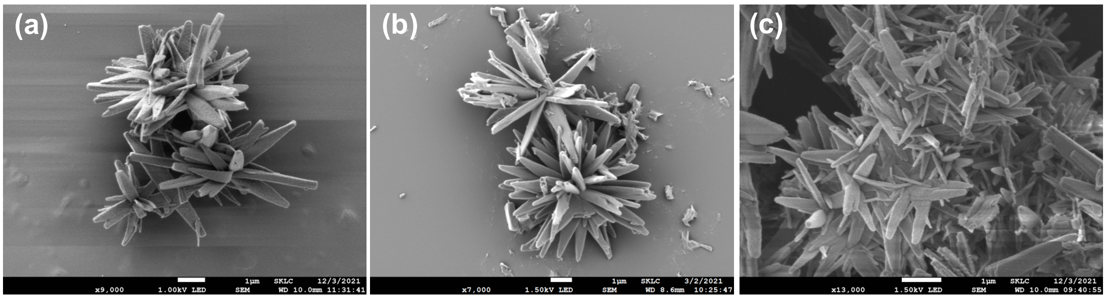

Supplementary Figure 7. Morphologies of COFs. SEM images of (a) CYANO-COF and (b) BD-COF after sonication for 30 min. SEM image of (c) CYANO-COF after sonication for 24 h. (scale bar: 1 μm)

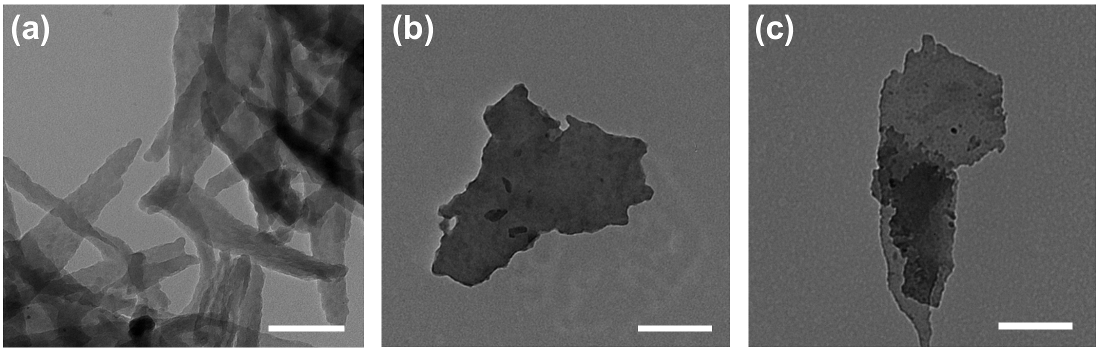

Supplementary Figure 8. TEM images. TEM images of (a) CYANO-COF, (b) CYANO-CON and (c) BD-CON (scale bar: 200 nm).

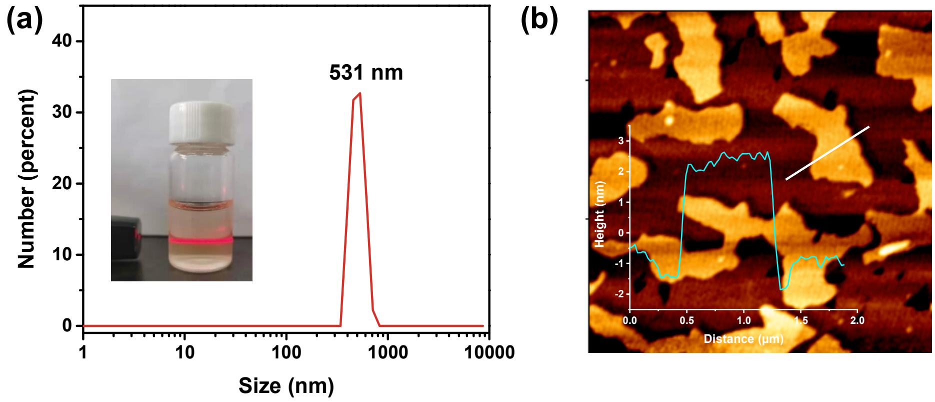

Supplementary Figure 9. Characterizations of BD-CON. (a) Particle size distribution by dynamic light scattering (inset: observed Tyndall effect) and (b) AFM image of BD-CON (inset: height plot).

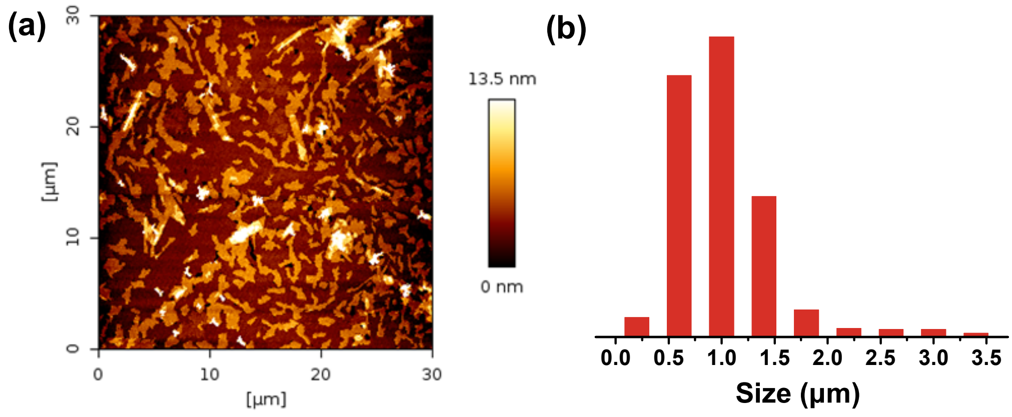

Supplementary Figure 10. Characterizations of CYANO-CON. (a) AFM image and (b) particle size distribution (measuring 200 particles) of CYANO-CON.

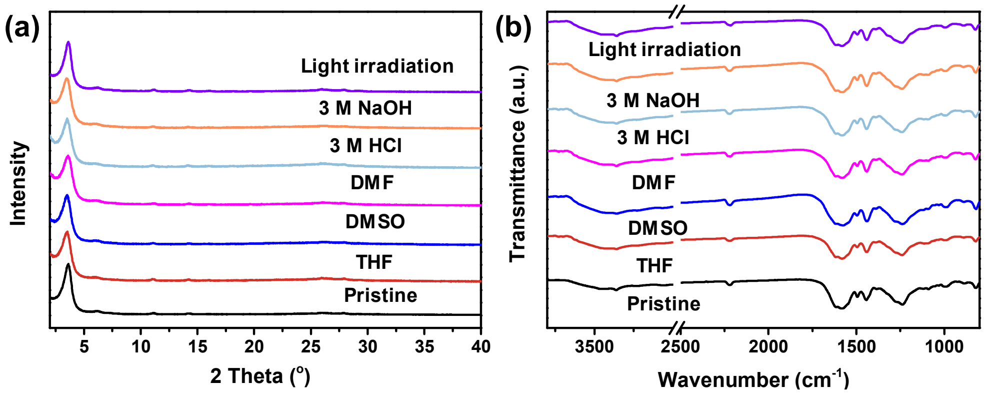

Supplementary Figure 11. Chemical stability of CYANO-COF. (a) PXRD patterns and (b) FT-IR spectra of CYANO-COF after treatment in 3 M NaOH, 3 M HCl, DMF, DMSO, THF and light irradiation in water for 3 days using a 300 W Xe lamp.

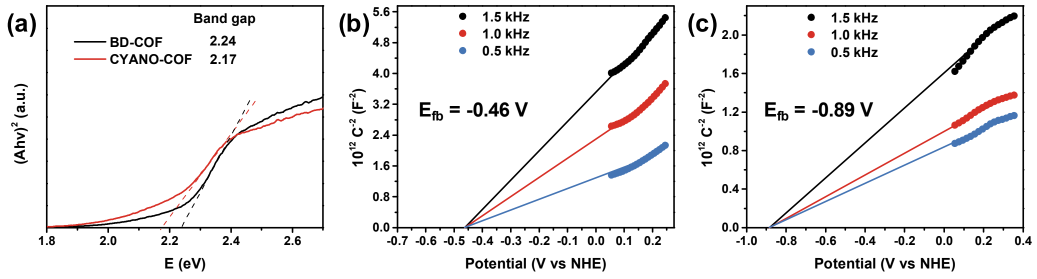

Supplementary Figure 12. Optical and electronic properties of COFs. (a) Tauc plots of BD-COF and CYANO-COF. Mott-Schottky plots for (b) BD-COF and (c) CYANO-COF in 0.2 M Na_2SO_4 aqueous solution with the photocatalyst-coated FTO as work electrode, a platinum foil as the counter electrode, and a saturated calomel electrode as the reference electrode at frequency of 0.5k, 1k and 2k Hz.

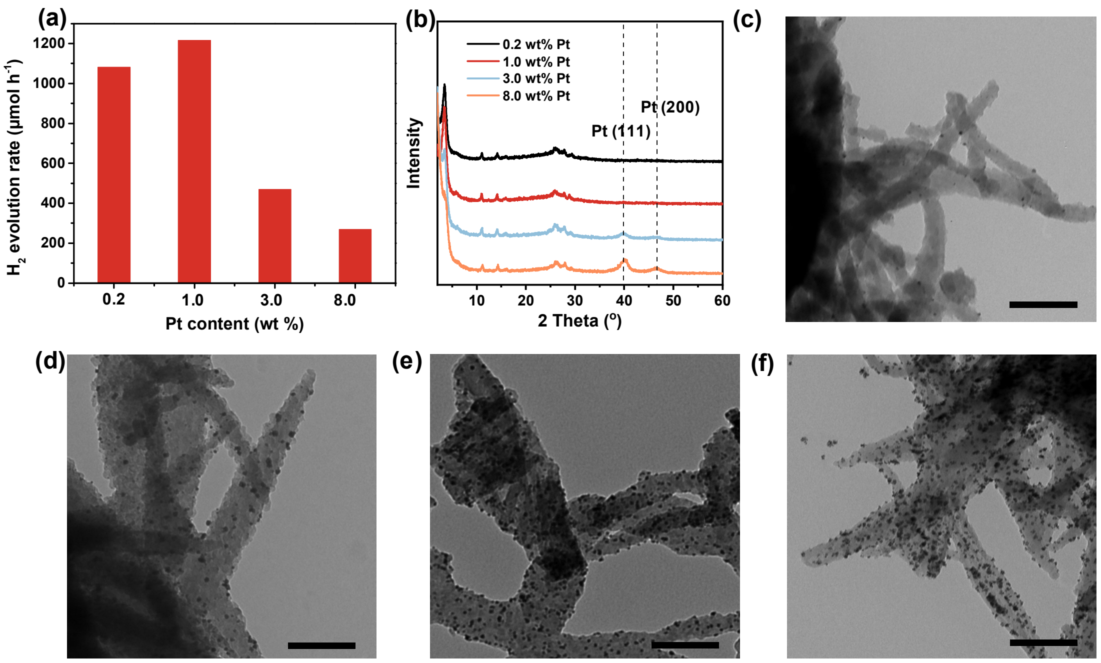

Supplementary Figure 13. Photocatalytic H_2 evolution. (a) Pt content dependent PHE activity of CYANO-COF, (b) PXRD patterns of CYANO-COF with different Pt content, TEM images of photo-deposition (c) 0.2 wt% Pt, (d) 1.0 wt% Pt, (e) 3.0 wt% Pt and (f) 8.0 wt% Pt over CYANO-COF (scale bar: 200 nm).

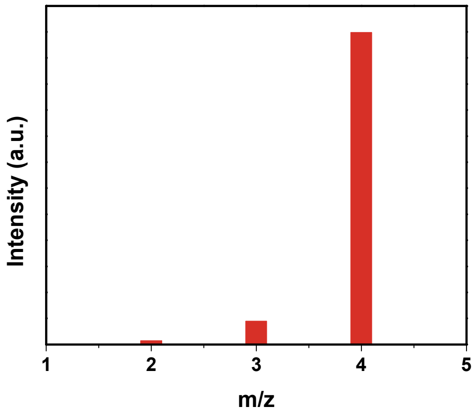

Supplementary Figure 14. Mass spectra analysis of evolved D_2 using D_2O as water source. Isotope labeling experiment was conducted by using D_2O instead of H_2O for photocatalytic hydrogen evolution which exhibits the evolution of D_2 gas.

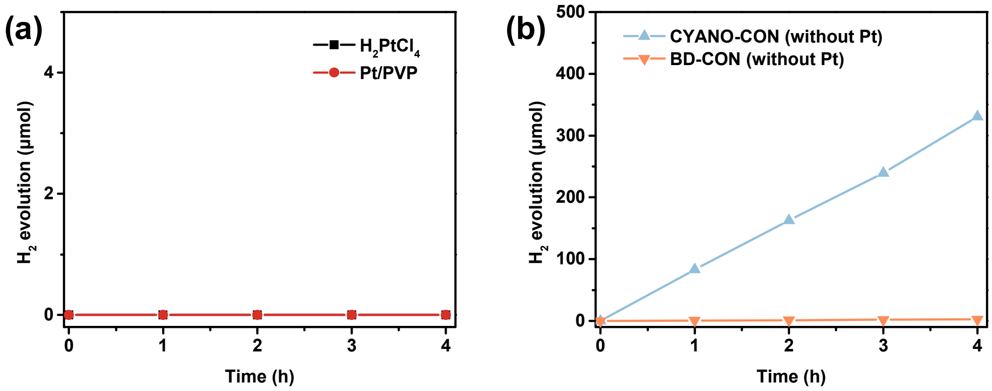

Supplementary Figure 15. Control experiments for PHE. Time course of photocatalytic H_2 production for blank control experiment with (a) 0.2 mg Pt/PVP or H_2PtCl_4, (b) 20 mg CONs without cocatalyst (100 mL water, 10 mmol ascorbic acid, $\lambda > 420$ nm).

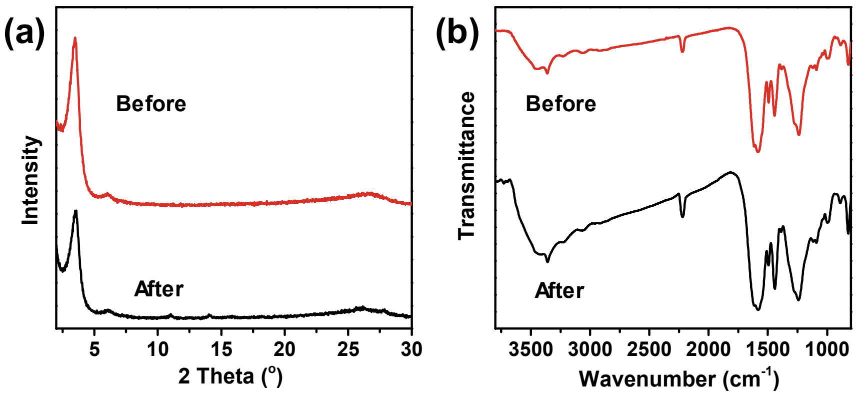

Supplementary Figure 16. Characterizations of CYANO-CON after cycling stability test. (a) PXRD pattern and (b) FT-IR spectrum of CYANO-CON before and after several catalytic cycles.

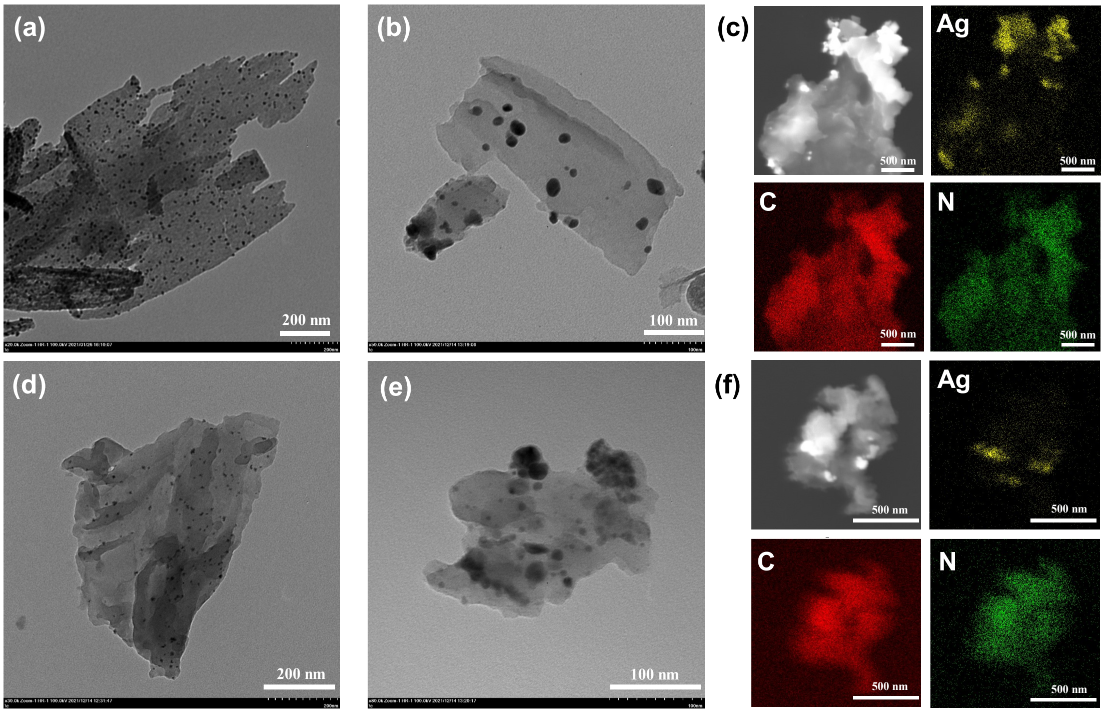

Supplementary Figure 17. TEM analysis of CONs after photocatalysis. (a,d) TEM images of (a) CYANO-CON and (d) BD-CON after HER, (b,e) TEM images of (b) CYANO-CON and (e) BD-CON after OER. (c,f) Elemental mapping of (c) CYANO-CON and (f) BD-CON after OER.

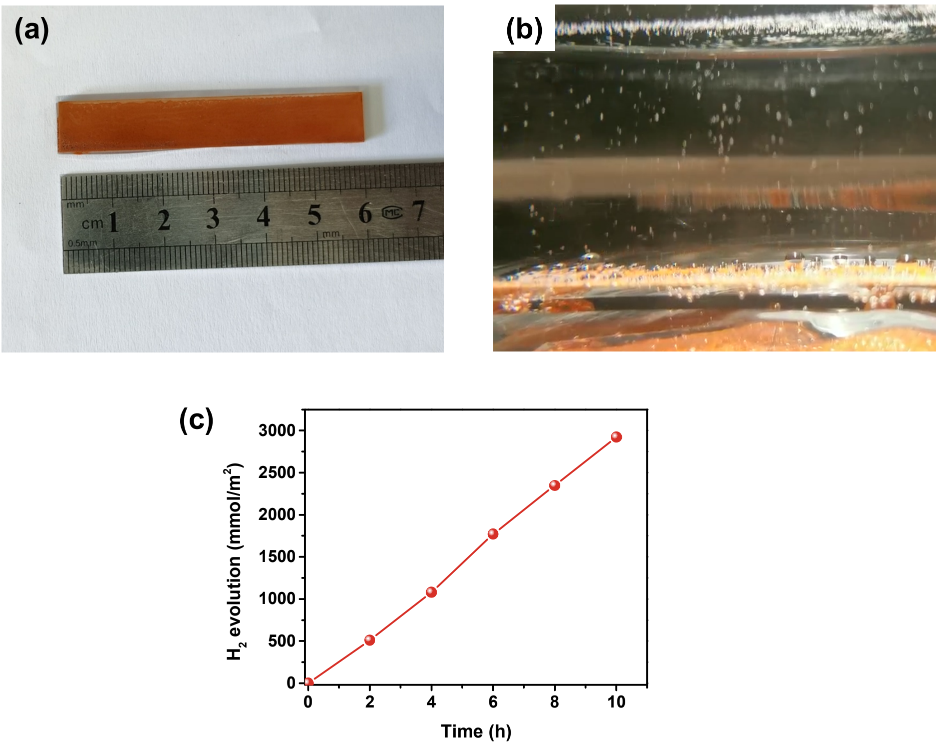

Supplementary Figure 18. PHE of CYANO-CON film on glass. (a) Photograph of as-prepared CYANO-CON film on glass, (b) photograph showing hydrogen evolution with CYANO-CON film on glass, (c) time course of photocatalytic H_2 evolution for CYANO-CON film on glass.

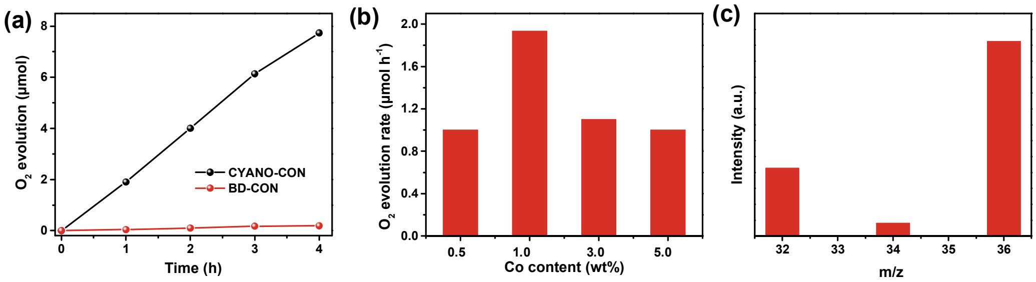

Supplementary Figure 19. Photocatalytic oxygen evolution. (a) Time course of photocatalytic O_2 production for CYANO-CON and BD-CON with 1wt % $Co(NO_3)_2$ as co-catalyst (20 mg catalyst in 100 mL water, 100 mg La_2O_3, 0.5 mmol $AgNO_3$, $\lambda >$ 420 nm). (b) Co content dependent oxygen evolution activity of CYANO-CON. (c) Isotope labeling experiment was conducted by using $H_2{}^{18}O$ instead of H_2O for photocatalytic oxygen evolution which exhibits the evolution of $^{18}O_2$ gas.

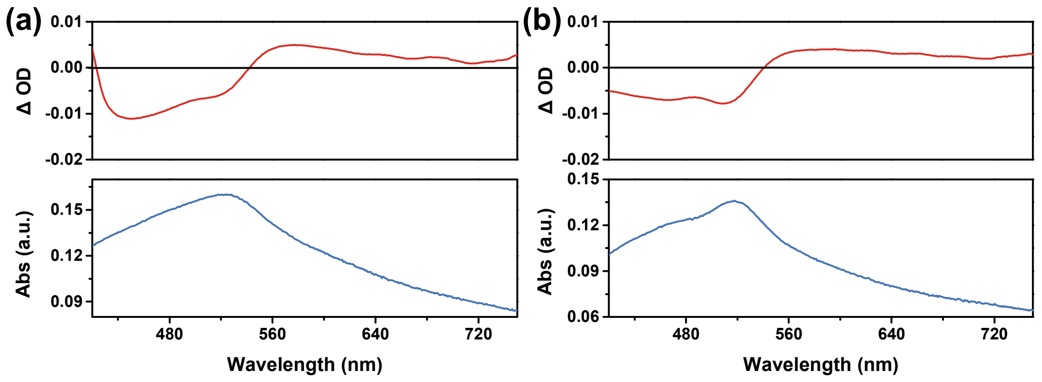

Supplementary Figure 20. TA and steady-state absorption spectra. TA spectra (red line) at 2 ps and steady-state absorption spectra (blue line) of (a) BD-CON and (b) CYANO-CON.

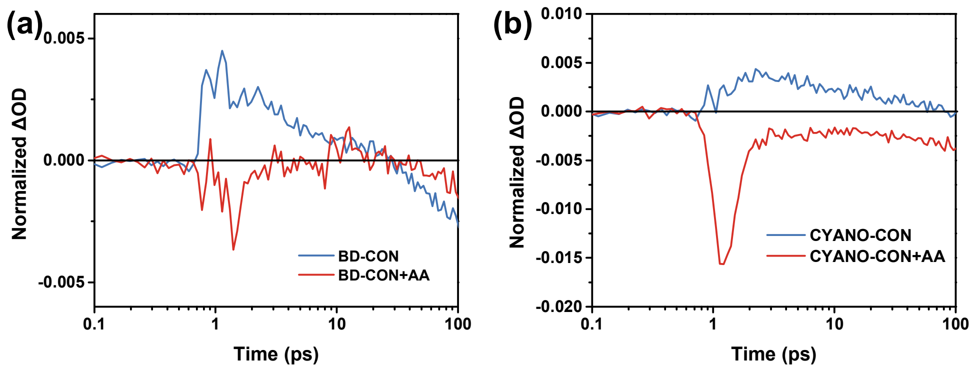

Supplementary Figure 21. TA kinetics of CONs. TA kinetics of (a) BD-CON and (b) CYANO-CON probed at 650 nm (trapped hole). In the presence of 0.1 M AA, the hole signal disappeared with the appearance of a negative absorption peak assigned to an ultra-fast component stemming from hole transfer from VB of CONs to the hole scavenger.

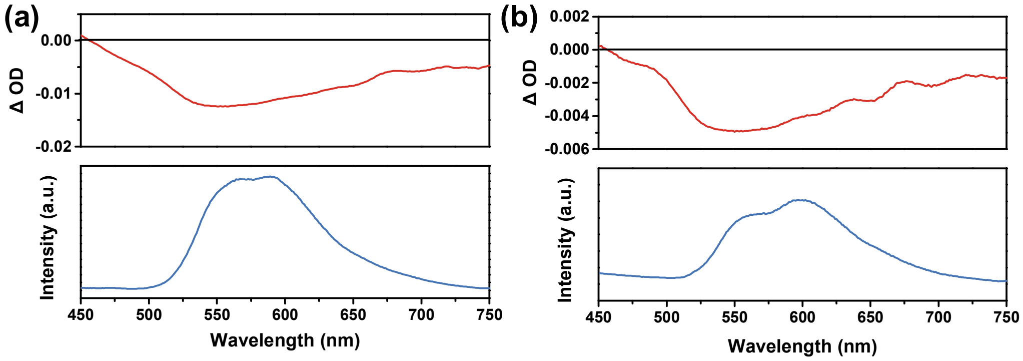

Supplementary Figure 22. TA and steady-state PL spectra. TA spectra (red line) at 1 ns and steady-state PL spectra (blue line) with excitation wavelength at 380 nm of (a) BD-CON and (b) CYANO-CON.

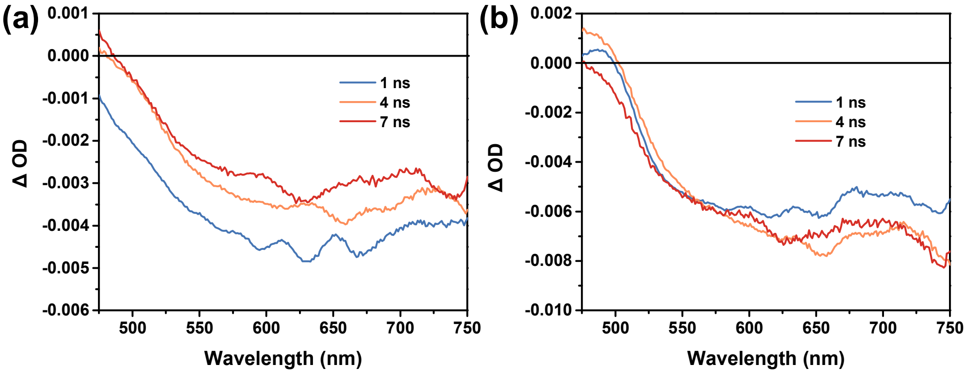

Supplementary Figure 23. TA analysis to illustrate the generation of long-lived free electrons. Time slices of the TA spectra of CYANO-CON in 0.1 M ascorbic acid of (a) BD-CON and (b) CYANO-CON. The negative signals can be attributed to long-lived electron in the presence of 0.1 M ascorbic acid.

(a)

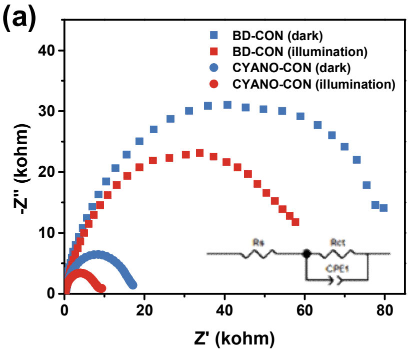

(b)

Sample	Rs/Ω	Rct/Ω
BD-CON (dark)	103.5	80169
BD-CON (illumination)	104.2	65910
CYANO-CON (dark)	102	17654
CYANO-CON (illumination)	101.8	8349

Supplementary Figure 24. EIS analysis. (a) Electrochemical impedance spectra of BD-CON and CYANO-CON were carried out under dark and illumination (> 420 nm, 15 A Xe lamp), with an AC potential frequency ranging from 0.1 Hz to 100 kHz. In the equivalent circuit (inset), R_s represents the circuit series-resistance, CPE1 is the capacitance phase element of the semiconductor-electrolyte interface, and R_{ct} is the charge transfer resistance across the interface, (b) Simulated R_s and R_{ct} values of CONs for electrochemical impedance test.

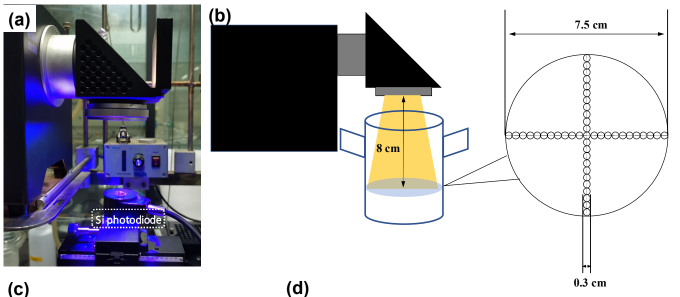

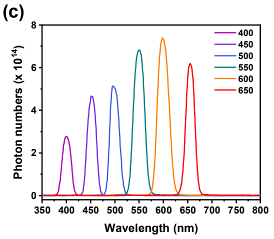

(d)

Wavelength (nm)	Number of incident photons per hour	H_2 evolution (μmol h^{-1})	AQE (%)
400	4.14×10^{20}	279.54	81.3
450	7.71×10^{20}	528.97	82.6
500	9.58×10^{20}	644.49	81.0
550	1.37×10^{21}	588.37	51.7
600	1.33×10^{21}	194.67	17.6
650	1.16×10^{21}	39.98	4.2

(e)

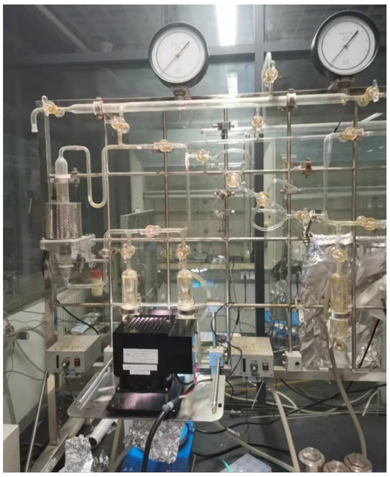

Supplementary Figure 25. Devices for AQE and OER. (a) Photographs of the devices used in the AQE measurement, (b) illustration of photon-counting system, (c) the relation of photon numbers and wavelength using different band-pass filters (measured at the center of light spot, diameter of 3 mm), (d) summary of the number of incident photons, PHE activity and AQE at different wavelengths, (e) the system for AQE and OER test.

Supplementary references

1. Kresse, G. & Furthmüller, J. Efficiency of ab-initio total energy calculations for metals and semiconductors using a plane-wave basis set. *Comput. Mater. Sci.* **6**, 15-50 (1996).

2. Kresse, G. & Furthmüller, J. Efficient iterative schemes for *ab initio* total-energy calculations using a plane-wave basis set. *Phys. Rev. B* **54**, 11169-11186 (1996).

3. Perdew, J. P., Burke, K. & Ernzerhof, M. Generalized gradient approximation made simple. *Phys. Rev. Lett.* **77**, 3865-3868 (1996).

4. Kresse, G. & Joubert, D. From ultrasoft pseudopotentials to the projector augmented-wave method. *Phys. Rev. B* **59**, 1758-1775 (1999).

5. Monkhorst, H. J. & Pack, J. D. Special points for Brillouin-zone integrations. *Phys. Rev. B* **13**, 5188-5192 (1976).

6. Takata, T. et al. Photocatalytic water splitting with a quantum efficiency of almost unity. *Nature* **581**, 411-414 (2020).

7. Zhao, Z. et al. Fabrication of robust covalent organic frameworks for enhanced visible-light-driven H_2 evolution. *ACS Catal.* **11**, 2098-2107 (2021).

8. Zhang, S. et al. Strong-base-assisted synthesis of a crystalline covalent triazine framework with high hydrophilicity via benzylamine monomer for photocatalytic water splitting. *Angew. Chem. Int. Ed.* **59**, 6007-6014 (2020).

9. Chen, W. et al. Modulating benzothiadiazole-based covalent organic frameworks via halogenation for enhanced photocatalytic water splitting. *Angew. Chem. Int. Ed.* **59**, 16902-16909 (2020).

10. Wang, X. et al. Sulfone-containing covalent organic frameworks for photocatalytic hydrogen evolution from water. *Nat. Chem.* **10**, 1180-1189 (2018).

11. Wei, S. et al. Semiconducting 2D triazine-cored covalent organic frameworks with unsubstituted olefin linkages. *J. Am. Chem. Soc.* **141**, 14272-14279 (2019).

12. Jin, E. et al. 2D sp^2 carbon-conjugated covalent organic frameworks for photocatalytic hydrogen production from water. *Chem* **5**, 1632-1647 (2019).

13. Sheng, J.-L. et al. Effect of different functional groups on photocatalytic hydrogen evolution in covalent-organic frameworks. *ChemCatChem* **11**, 2313-2319 (2019).

14. Liu, M. et al. Crystalline covalent triazine frameworks by in situ oxidation of alcohols to aldehyde monomers. *Angew. Chem. Int. Ed.* **57**, 11968-11972 (2018).

15. Bi, S. et al. Two-dimensional semiconducting covalent organic frameworks via condensation at arylmethyl carbon atoms. *Nat. Commun.* **10**, 2467 (2019).
16. Xu, J. et al. Vinylene-linked covalent organic frameworks (COFs) with symmetry-tuned polarity and photocatalytic activity. *Angew. Chem. Int. Ed.* **59**, 23845-23853 (2020).
17. Guo, L., Niu, Y., Razzaque, S., Tan, B. & Jin, S. Design of D−A_1−A_2 covalent triazine frameworks via copolymerization for photocatalytic hydrogen evolution. *ACS Catal.* **9**, 9438-9445 (2019).
18. Chen, R. et al. Rational design of isostructural 2D porphyrin-based covalent organic frameworks for tunable photocatalytic hydrogen evolution. *Nat. Commun.* **12**, 1354 (2021).
19. Mi, Z. et al. Covalent organic frameworks enabling site isolation of viologen-derived electron-transfer mediators for stable photocatalytic hydrogen evolution. *Angew. Chem. Int. Ed.* **60**, 9642-9649 (2021).
20. Yin, L. et al. Structure-property relationship in β-keto-enamine-based covalent organic frameworks for highly efficient photocatalytic hydrogen production. *Chem. Eng. J.* **419**, 129984 (2021).
21. Yang, J. et al. Protonated imine-linked covalent organic frameworks for photocatalytic hydrogen evolution. *Angew. Chem. Int. Ed.* **60**, 19797-19803 (2021).
22. Wang, D. et al. Highly efficient charge transfer in CdS-covalent organic framework nanocomposites for stable photocatalytic hydrogen evolution under visible light. *Sci. Bull.* **65**, 113-122 (2020).
23. Li, C.-C. et al. Rational combination of covalent-organic framework and nano TiO_2 by covalent bonds to realize dramatically enhanced photocatalytic activity. *Appl. Catal. B* **266**, 118586 (2020).
24. Luo, M., Yang, Q., Liu, K., Cao, H. & Yan, H. Boosting photocatalytic H_2 evolution on g-C_3N_4 by modifying covalent organic frameworks (COFs). *Chem. Commun.* **55**, 5829-5832 (2019).
25. Gao, M.-Y. et al. Boosting visible-light-driven hydrogen evolution of covalent organic frameworks through compositing with MoS_2: a promising candidate for noble-metal-free photocatalysts. *J. Mater. Chem. A* **7**, 20193-20200 (2019).
26. Thote, J. et al. A covalent organic framework-cadmium sulfide hybrid as a prototype photocatalyst for visible-light-driven hydrogen production. *Chem. Eur. J.* **20**, 15961-15965 (2014).

27. Ming, J. et al. Hot π-electron tunneling of metal-insulator-COF nanostructures for efficient hydrogen production. *Angew. Chem. Int. Ed.* **58**, 18290-18294 (2019).
28. Luo, M. et al. Defects engineering leads to enhanced photocatalytic H_2 evolution on graphitic carbon nitride-covalent organic framework nanosheet composite. *Small* **16**, 2001100 (2020).
29. Zhou, T. et al. PEG-stabilized coaxial stacking of two-dimensional covalent organic frameworks for enhanced photocatalytic hydrogen evolution. *Nat. Commun.* **12**, 3934 (2021).
30. Xing, Y. et al. Construction of the 1D covalent organic framework/2D g-C_3N_4 heterojunction with high apparent quantum efficiency at 500 nm. *ACS Appl. Mater. Interfaces* **12**, 51555-51562 (2020).
31. Liu, L. et al. Supercritical CO_2 produces the visible-light-responsive TiO_2/COF heterojunction with enhanced electron-hole separation for high-performance hydrogen evolution. *Nano Res.* **13**, 983-988 (2020).
32. Shu, C. et al. Boosting the photocatalytic hydrogen evolution activity for D–π–A conjugated microporous polymers by statistical copolymerization. *Adv. Mater.* **33**, 2008498 (2021).
33. Sprick, R. S. et al. Photocatalytic hydrogen evolution from water using fluorene and dibenzothiophene sulfone-conjugated microporous and linear polymers. *Chem. Mater.* **31**, 305-313 (2019).
34. Sachs, M. et al. Understanding structure-activity relationships in linear polymer photocatalysts for hydrogen evolution. *Nat. Commun.* **9**, 4968 (2018).
35. Shu, G., Li, Y., Wang, Z., Jiang, J.-X. & Wang, F. Poly(dibenzothiophene-S,S-dioxide) with visible light-induced hydrogen evolution rate up to 44.2 mmol h^{-1} g^{-1} promoted by K_2HPO_4. *Appl. Catal. B* **261**, 118230 (2020).
36. Valverde-González, A. et al. Understanding charge transfer mechanism on effective truxene-based porous polymers–TiO_2 hybrid photocatalysts for hydrogen evolution. *ACS Appl. Energy Mater.* **3**, 4411-4420 (2020).
37. Aitchison, C. M., Sprick, R. S. & Cooper, A. I. Emulsion polymerization derived organic photocatalysts for improved light-driven hydrogen evolution. *J. Mater. Chem. A* **7**, 2490-2496 (2019).
38. Chen, J. et al. Novel conjugated organic polymers as candidates for visible-light-driven photocatalytic hydrogen production. *Appl. Catal. B* **241**, 461-470 (2019).
39. Shu, G. et al. A high performance and low cost poly(dibenzothiophene-S,S-

dioxide)@TiO_2 composite with hydrogen evolution rate up to 51.5 mmol h^{-1} g^{-1}. *J. Mater. Chem. A* **8**, 18292-18301 (2020).
40. Zhang, X., Peng, B., Zhang, S. & Peng, T. Robust wide visible-light-responsive photoactivity for H_2 production over a polymer/polymer heterojunction photocatalyst: the significance of sacrificial reagent. *ACS Sustainable Chem. Eng.* **3**, 1501-1509 (2015).
41. Chen, L., Xu, Y., Yang, Z., Zhang, K. & Chen, B. Cobalt (II)-based open-framework systems constructed on g-C_3N_4 for extraordinary enhancing photocatalytic hydrogen evolution. *Appl. Catal. B* **277**, 119207 (2020).
42. She, X. et al. High efficiency photocatalytic water splitting using 2D α-Fe_2O_3/g-C_3N_4 Z-scheme catalysts. *Adv. Energy Mater.* **7**, 1700025 (2017).
43. Zhao, C., Ding, C., Han, C., Yang, X. & Xu, J. Lignin-incorporated supramolecular copolymerization yielding g-C_3N_4 nanoarchitectures for efficient photocatalytic hydrogen evolution. *Sol. RRL* **5**, 2000486 (2021).
44. Han, Q. et al. Atomically thin mesoporous nanomesh of graphitic C_3N_4 for high-efficiency photocatalytic hydrogen evolution. *ACS Nano* **10**, 2745-2751 (2016).
45. Yu, Y. et al. Surface engineering for extremely enhanced charge separation and photocatalytic hydrogen evolution on g-C_3N_4. *Adv. Mater.* **30**, 1705060 (2018).
