# Supplementary material for: Covalent organic frameworks with high quantum efficiency in sacrificial photocatalytic hydrogen evolution
Source: Nat Commun. 2022 Apr 29;13:2357. doi: 10.1038/s41467-022-30035-x (PMC9054748; doi:10.1038/s41467-022-30035-x)
Supplement: Supplementary file 3 — Description of Additional Supplementary Files [file 41467_2022_30035_MOESM3_ESM.pdf]

Description of Additional Supplementary Files

File Name: Supplementary Movie 1
Description: CYANO-CON film irradiated under visible light (λ > 420 nm).
